# Nuclear magnetic resonance spectroscopy to quantify major extracellular matrix components in fibro-calcific aortic valve disease

**DOI:** 10.1038/s41598-023-46143-7

**Published:** 2023-11-01

**Authors:** Lukas Feistner, Anja Penk, Julia Böttner, Petra Büttner, Holger Thiele, Daniel Huster, Florian Schlotter

**Affiliations:** 1 Department of Internal Medicine/Cardiology, Heart Center Leipzig at University of Leipzig, Struempellstr. 39, 04289 Leipzig, Germany; 2 Institute for Medical Physics and Biophysics, Leipzig University, Leipzig, Germany

**Keywords:** Biochemistry, Biophysics, Cell biology, Cardiology

## Abstract

Fibro-calcific aortic valve disease (FCAVD) is a pathological condition marked by overt fibrous and calcific extracellular matrix (ECM) accumulation that leads to valvular dysfunction and left ventricular outflow obstruction. Costly valve implantation is the only approved therapy. Multiple pharmacological interventions are under clinical investigation, however, none has proven clinically beneficial. This failure of translational approaches indicates incomplete understanding of the underlying pathomechanisms and may result from a limited toolbox of scientific methods to assess the cornerstones of FCAVD: lipid deposition, fibrous and calcific ECM accumulation. In this study, we evaluated magic-angle spinning (MAS) nuclear magnetic resonance (NMR) spectroscopy to both, qualitatively and quantitatively assess these key elements of FCAVD pathogenesis. NMR spectra showed collagen, elastin, triacylglycerols, and phospholipids in human control and FCAVD tissue samples (n = 5). Calcification, measured by the hydroxyapatite content, was detectable in FCAVD tissues and in valve interstitial cells under procalcifying media conditions. Hydroxyapatite was significantly higher in FCAVD tissues than in controls (p < 0.05) as measured by ^31^P MAS NMR. The relative collagen content was lower in FCAVD tissues vs. controls (p < 0.05). Overall, we demonstrate the versatility of NMR spectroscopy as a diagnostic tool in preclinical FCAVD assessment.

## Introduction

Fibro-calcific aortic valve disease (FCAVD) has a high prevalence especially in the aging population in the western world^1^. Lipid and phospholipid deposition, immune cell infiltration and inflammation, and myofibroblastic as well as osteogenic differentiation of resident valve interstitial cells (VICs) are the underlying pathomechanisms^2–4^. Myofibroblastic VICs actively trigger extracellular matrix (ECM) remodelling with collagen accumulation, valvular thickening and tissue calcification^5^. Proteoglycan degeneration and elastic fibre fragmentation further contribute to the alterations in the valvular architecture^6,7^. These structural changes in the connective tissue cause major hemodynamic impairment, culminating in aortic valve stenosis (AS) that associates with heart failure and substantially increases mortality^8,9^. Costly aortic valve (AV) implantation is available, however, limited valve durability, procedural risk and unsuitable patient characteristics are important factors that limit the clinical application^10^. Although progress in the mechanistic understanding of the FCAVD pathogenesis has been made, a pharmacological treatment is missing^11^. In recent years, preclinical in vivo and in vitro models implicated various promoting factors and molecular pathways in FCAVD^12^.

Elevated phosphate concentrations in the blood serum are associated with increased valvular calcification, indicating the important role of phosphate in FCAVD^13,14^. Therefore, in this study, a procalcifying medium (PM), containing inorganic phosphate, was used to induce passage independent calcification in VIC cultures^15^. However, it is not completely clear whether this in vitro modelling mirrors the complex, long-lasting in vivo FCAVD pathomechanisms. In addition, established analytical tools only depict individual molecular components like collagen, elastin, and lipids, or indicate calcification. Thus, a multitude of analytic methods is necessary for a comprehensive assessment of a single FCAVD progression stage.

Solid-state nuclear magnetic resonance (NMR) spectroscopy under magic-angle spinning (MAS) conditions acquires spectra from solid materials by direct excitation or cross polarization (CP) and identifies mobile or rigid molecules of a sample, respectively^16^. ^13^C MAS NMR allows the analysis of ECM organic compounds such as collagen, elastin, phospholipids and triglycerides^17–20^, while ^31^P NMR assesses calcification. Thus, NMR spectroscopy may represent a versatile tool to investigate major FCAVD pathomechanistic characteristics in a native specimen at once. Hence, this study focusses on the analysis of the relevant major organic and inorganic components in FCAVD tissues as well as in in vitro models using ^13^C and ^31^P MAS NMR. ECM quantification using conventional histochemical methods is challenging because of its restriction to exemplary two-dimensional tissue sections. Thus, special attention was payed to the ECM quantification in vivo and in vitro using NMR spectra.

## Results

### ^13^C Solid-state NMR of organic aortic valve components and organic cell culture material

**Figure 1:**
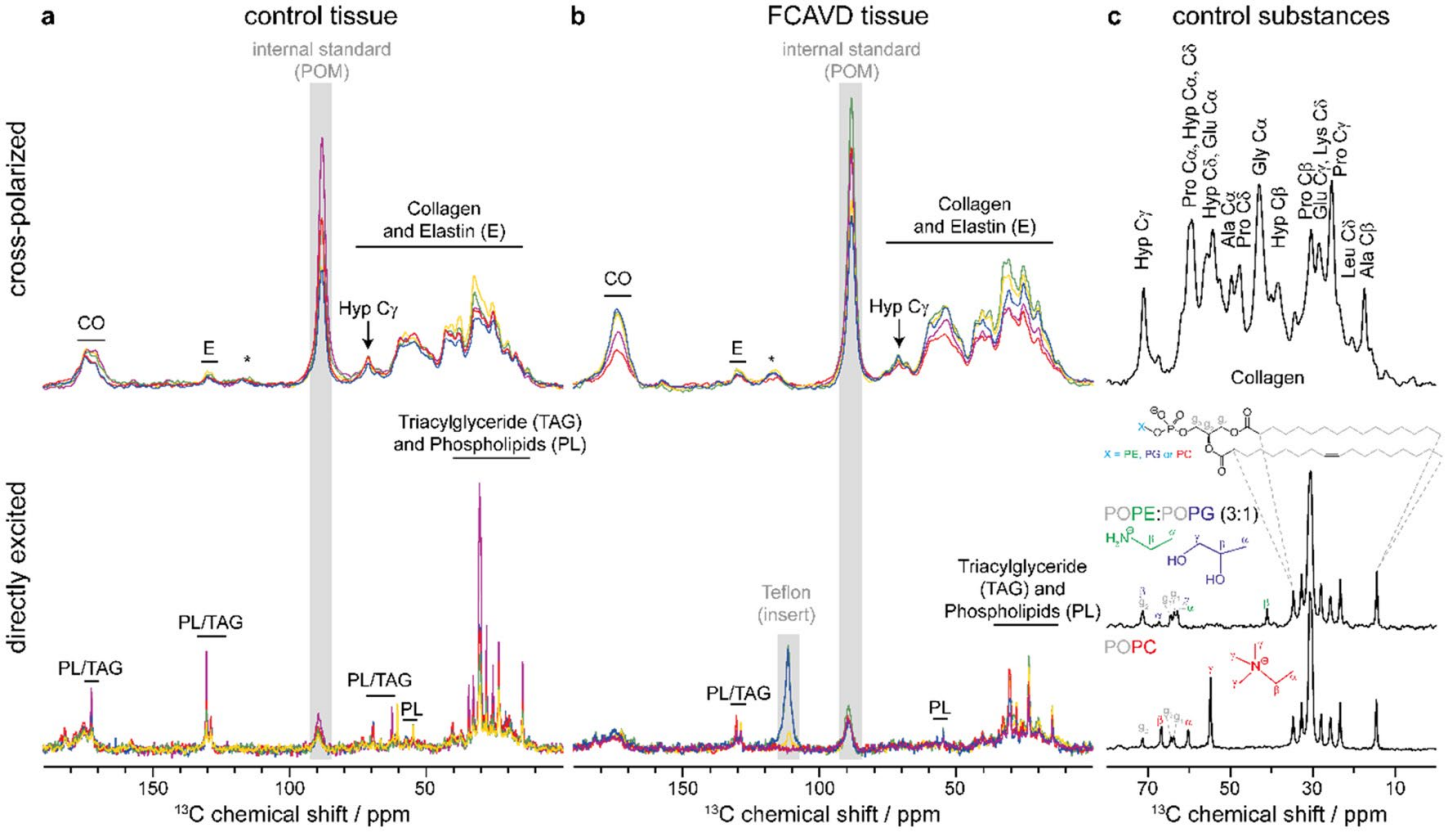
Detection of collagen, elastin, triacylglycerols and phospholipids by ^13^C MAS NMR spectroscopy in (**a**) control tissue and (**b**) fibro-calcific aortic valve disease (FCAVD) tissue. Top panel shows cross-polarized NMR spectra and the lower panel the respective directly excited NMR spectra for n = 5 specimen per group. Spectra are scaled per mg wet sample weight. Control spectra of the aliphatic region of collagen and different phospholipid membranes are shown in panel (**c**). Signals from internal standard (POM: polyoxymethylene) or the Teflon insert of the MAS rotor are indicated in grey, asterisks denote spinning sidebands (from CO signal). *CO* carbonyl group, *E* elastin, *Hyp* *C*_*γ*_ hydroxyproline C_γ_, *PL* phospholipids, *TAG* triacylglycerols.

To characterize the ECM composition in healthy control tissue (CT) and end-stage FCAVD tissues, solid-state ^13^C NMR spectroscopy was performed (Fig. 1). ^13^C CP MAS NMR spectra are sensitive to the immobile components of the ECM and predominantly identified collagen (Fig. 1, upper panel).

In the directly excited ^13^C NMR spectra, the signals of phospholipids (PL) and triacylglycerols (TAG) were dominating, representing a superposition of both compounds. As only the chemical environment influences the chemical shift, the lipid chains of the PLs and TAGs exhibit the same fingerprint in the aliphatic range between 10 and 40 ppm as well as for the unsaturated hydrocarbon (130 ppm) and glycerol signals (see Fig. 1c, gray). However, different headgroups of PLs can be distinguished depending on their chemical shift, e.g., phosphatidylethanolamine (PE, green), phosphatidylglycerol (PG, purple) and phosphatidylcholine (PC, red) in Fig. 1c. Only a minor peak of the characteristic methyl groups (Cγ) of the PC head group was identified around 55 ppm (Fig. 1). Hence, PCs did not represent the majority of the detected PLs and TAGs. Furthermore, an overall trend of higher PL and TAG content in the CT was observed compared to FCAVD tissue as indicated by higher signal intensities in the aliphatic region, of the double bond signal (130 ppm) as well as of the CO signal in the CT spectra.

**Figure 2:**
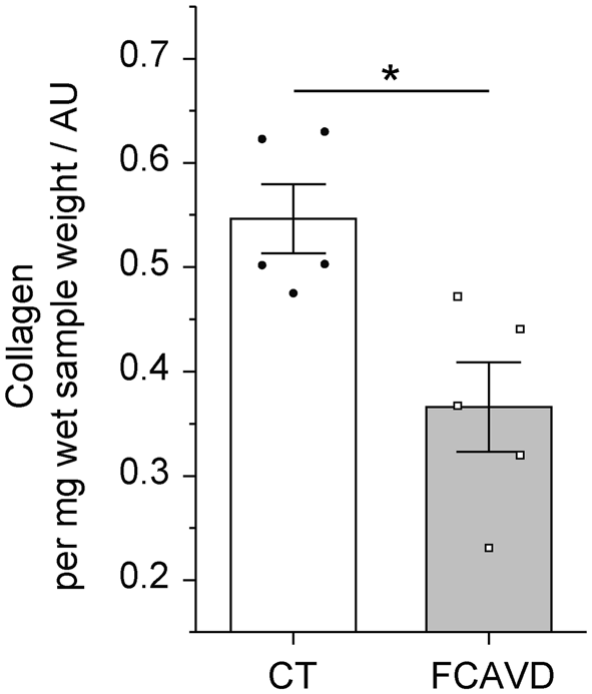
Quantitative analysis of the collagen content in aortic valve tissues based on integration of characteristic NMR signals. Quantification of hydroxyproline content (representative for the collagen amount) in control (CT) and fibro-calcific aortic valve disease (FCAVD) tissues. n = 5; *p < 0.01, error bars—standard error.

Employing the hydroxyproline C_γ_ (Hyp C_γ_) intensity and the internal standard polyoxymethylene (POM) as well as the sample weight in the CP spectra, the relative collagen amount per mg sample was determined. It was decreased in FCAVD tissues compared to CT (Fig. 2; CT: 0.55 ± 0.03 AU, FCAVD: 0.37 ± 0.04 AU; p < 0.05, n = 5).

In parallel, VIC in vitro cultures were analyzed by ^13^C MAS NMR. Directly excited NMR spectra revealed low signal to noise ratio (SNR). The ^13^C CP NMR spectra showed no specific signals for collagen (Suppl. Fig. 1), which is in contrast to FCAVD tissues. Two PM-treated VIC cultures showed a prominent signal around 30 ppm (green and red line, Suppl. Fig. 1). This signal is assigned to chemically equivalent CH_2_ groups of fatty acids^21^. However, the broad line shape and the good CP sensitivity indicated restricted motion of the fatty acids.

All other samples had lower signal intensities and a low SNR in the ^13^C NMR spectra and were not further analyzed as optimization of the CP step, which crucially influences the observed peak intensity, is not possible without sufficient signal intensity.

### ^31^P solid-state NMR of inorganic components in aortic valves and cell culture

**Figure 3:**
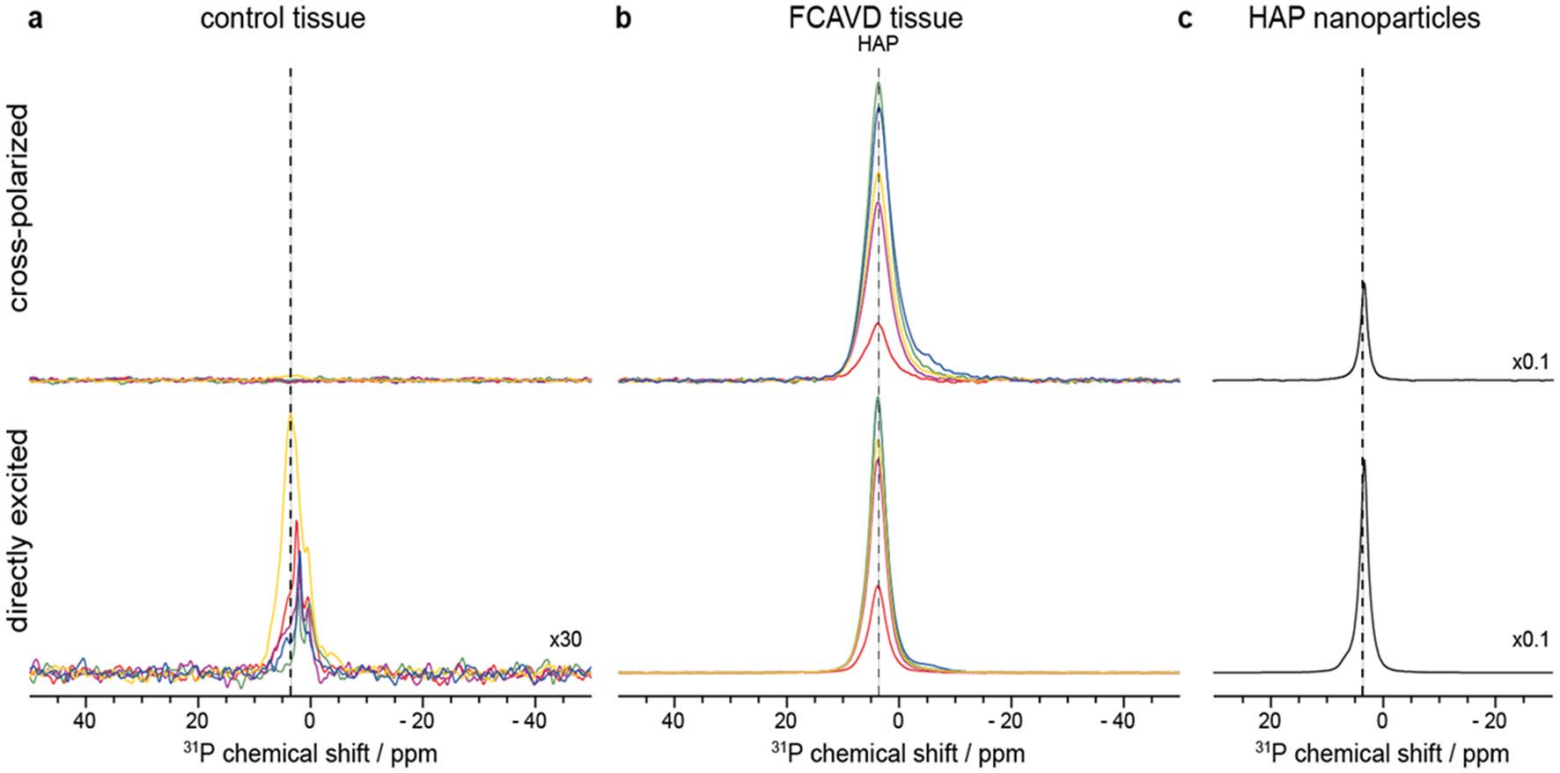
Detection of hydroxyapatite (HAP) in tissue samples using ^31^P MAS NMR spectra, upper panels show cross polarized, lower panels directly excited ^31^P NMR spectra of (**a**) control tissue (CT), (**b**) fibro-calcific aortic valve disease (FCAVD) tissue, and **c** HAP nanoparticles as control. Spectra are scaled per mg wet sample, except CT tissue in the lower panel, which is 30 times magnified. Spectra of HAP nanoparticles are scaled by a factor of 0.1. Chemical shift of HAP is indicated by dashed line; n = 5 per group.

^31^P MAS NMR was used to identify and characterize calcification in FCAVD tissues and PM-treated VICs. Direct excitation detects all phosphorous species in the sample, whereas CP NMR spectra detect only signals from rigid phosphorous moieties in close proximity to hydrogens. As for ^13^C, the chemical shift of ^31^P is highly sensitive to the chemical environment. Taken together, this allows to assess the degree of calcification of the specimen, as hydroxyapatite (HAP) can be identified using both spectra and quantified in the directly excited spectrum (see Fig. 3c for reference). In FCAVD tissues, distinct ^31^P NMR spectra representing HAP were detected in all samples (by direct excitation and CP, Fig. 3b).

**Figure 4:**
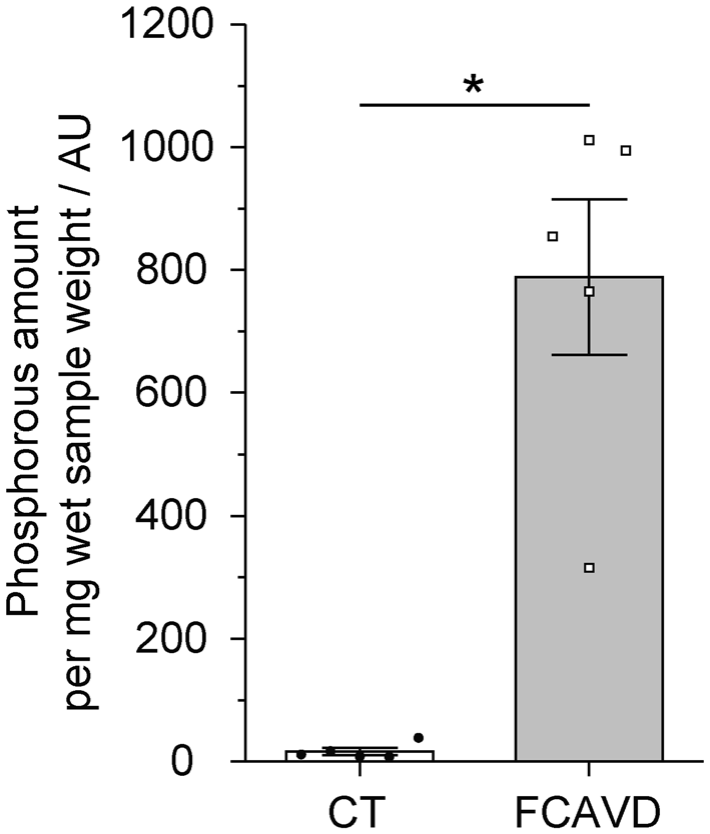
Quantitative analysis of the phosphate content in aortic valve tissues based on integration of NMR signals. Quantification of the phosphate amount of control (CT) and fibro-calcific aortic valve disease (FCAVD) tissues. n = 5; *p < 0.01, error bars—standard error.

A minor phosphorous signal, which was, for most specimen, slightly shifted compared to the HAP signal in FCAVD tissue (dashed line) and therefore does not represent HAP, was observed in directly excited NMR spectra of CT (Fig. 3a, lower panel, 30 times magnified). Furthermore, none of these signals were cross polarizable, indicating the lack of nearby protons. Thus, no HAP was detected in CP NMR spectra (Fig. 3) for CT tissue. NMR spectral integral of the directly excited spectra was used to calculate and compare the amount of mineralization between FCAVD and CT (Fig. 4, CT: 16 ± 6 AU, FCAVD: 788 ± 126 AU; p < 0.05). The uncertainties due to imperfections of pulse calibration and integration were determined to be below 5% (see “Materials and methods”).

**Figure 5:**
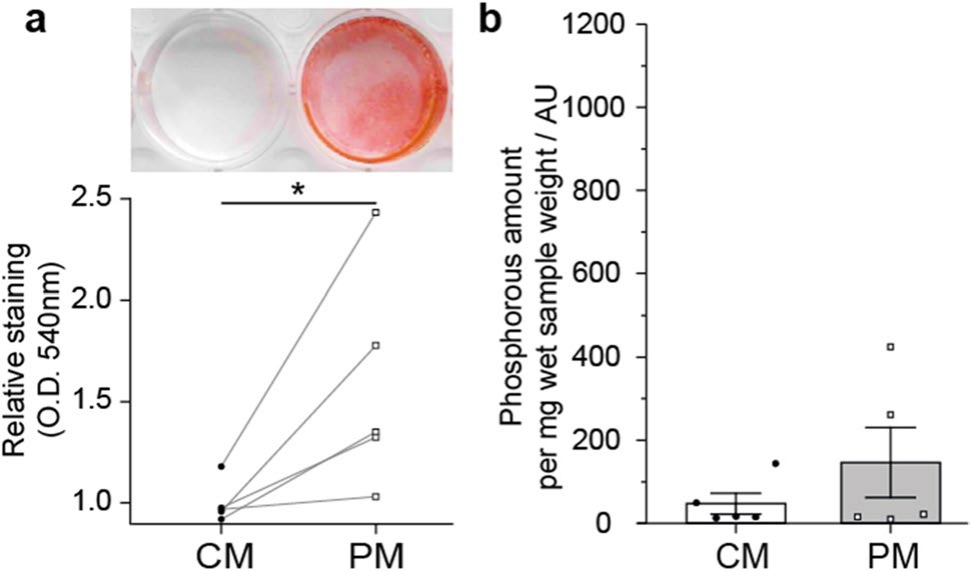
Quantification of mineralisation of valve interstitial cells treated with pro-calcifying medium to induce calcification. (**a**) Alizarin red staining for quantification of mineralization of valvular interstitial cells after treatment for 21 days with control medium (CM) or pro-calcifying medium (PM) and (**b**) quantification by ^31^P NMR. x-axis is scaled according to Fig. 4, to allow easy comparison between tissue and cell culture. n = 5; *p < 0,05.

PM treatment reproducibly induced calcific mineral deposition as quantified by Alizarin Red in the VIC in vitro model (Fig. 5a, p < 0.05). However, ^31^P MAS NMR provided a weak signal intensity in PM-treated VICs, except for two specimen (green and red colour, Suppl. Fig. 2) which also had a calcified fatty acid signal in the ^13^C CP NMR spectra (see above). Furthermore, one control medium (CM)-treated sample showed a low HAP signal. The quantification of the phosphorous amount in all experimental groups was comparable (Fig. 5b).

## Discussion

NMR spectroscopy has been used for the analysis of a multitude of biological tissues^17–20,22^ especially in vascular calcification, which shares features with FCAVD^23,24^. In this study, we demonstrate, for the first time, that NMR spectroscopy is eminently suitable for the detection and quantification of major components of the valvular ECM in vivo in non-diseased and diseased aortic valves as well as in VIC in vitro cultures. In particular, this NMR-based analysis revealed and quantified:

1. Triglycerides, phospholipids, collagen and elastin in valvular CT and FCAVD tissues.
2. Increased HAP content and lower collagen content in FCAVD tissues compared to controls.
3. Fatty acids undergoing restricted motion on the NMR timescale in PM-treated VIC cultures.

Mineralized and non-mineralized ECM components in aortic valve tissue are frequently analyzed by histochemistry, immunohistochemistry, electron microscopy, infrared spectroscopy, and x-ray-based techniques^25–28^. These methods detect particular ECM components, but are limited in their applicability to screen and quantify multiple ECM elements in parallel.

NMR spectroscopy has the distinct advantage that biological tissue can be investigated without the need for fixation, staining or other treatments. Organic and inorganic components are analyzed within the same sample by ^13^C and ^31^P MAS NMR spectroscopy at once^17^ allowing for a qualitative and quantitative characterization of major ECM components. Accordingly, lipids and phospholipids, collagen, elastin and HAP, key players in FCAVD pathophysiology^17,29^, were identified using NMR. Collagen was inversely correlated with HAP content. While calcification was increased in FCAVD, the collagen content per mg wet sample weight was decreased compared to CT. Although these results are consistent with prior data suggesting collagen degradation as well as calcific mineral accumulation in FCAVD^30,31^, the total collagen per leaflet might still be increased in FCAVD relative to CT^32^. Regarding the role of lipids in FCAVD pathophysiology, accumulating evidence suggests that oxidized phospholipids carried by lipoprotein a, may initiate a complex inflammatory process leading to aortic valve calcification^33,34^. Interestingly, we observed that the relative content of lipids, especially TAGs, was slightly reduced in FCAVD compared to healthy CT.

NMR spectroscopy on PM-treated VICs in vitro proved that HAP formation is evident as well as in in vivo calcification^25^. Thus, PM treatment might represent a sufficient calcification model to reproduce valvular ECM mineralization in vitro. Nevertheless, in this study two of five samples showed HAP formation as detected by ^31^P MAS NMR, whereas Alizarin red staining detected calcification in all samples. Natural variability in human primary cell cultures, patient-specific variability and the staining of all calcium containing mineral material by Alizarin red may have caused this discrepancy^13^.

Surprisingly, the NMR method did not detect glycosaminoglycans (GAGs), which are considered to represent a major component of the spongiosa layer^25^, suggesting that GAG concentration is at least ten fold below the readily detectable lipids/TAGs. While the GAG content may be below the detection limit in CT, the missing signal is surprising for FCAVD tissue, where excess production of GAGs and proteoglycans has been described^29,35^.

Co-detection of collagen and elastin, both important ECM components of the AV, by NMR, is rather difficult, since it is known that NMR spectra of elastin are strongly influenced by the water content^36^. Due to the water uptake of isolated elastin, spectral intensity increases at lower humidity^36^. Hence, the detection of elastin was more straightforward in the FCAVD tissue due to its lower water content secondary to the higher calcific mineral content. Regarding the quantification of collagen, the hydroxyproline Cγ (Hyp C_γ_) resonance around 71 ppm is used, as hydroxyproline content of collagen is roughly 13%. However, elastin also contains 1–2% of hydroxyproline^37,38^ and is about 3.4 times less abundant than collagen in the AV^32^. Hence, a maximum of 4.3% of the detected hydroxyproline may originate from elastin instead of collagen and this may result in a slight overestimation of the collagen content. However, as this is within the margins of error for quantification using NMR and holds true for both, control and FCAVD tissues, we used the Hyp C_γ_ signal for the semi-quantitative assessment of the collagen content.

In this study, an inconsistent detection of HAP by NMR compared to reproducible detection by Alizarin red was observed. The extraction method used for NMR detection may not be sufficient to extract the firmly bound HAP from the cell culture dish. Hence, harsher extraction methods may be used to optimize the extraction yield of significantly mineralized VIC matrices. Harsher extraction, of more firmly bound matrix generated by the cells treated with PM, resulted in small remnants of plastic, not visible by eye, produced during the scratching procedure. These plastic impurities, from the cell culture dish, produced high intensity NMR peaks, which hindered adequate interpretation of NMR signals in the ^13^C CP spectra and the calculation of relative amounts, as the plastic impurities also contribute to the sample weight.

In summary, NMR analyses require a high amount of sample material, which is suitable for tissue biopsy analyses, but demands large cell cultures with adapted extraction methods. Applying additional ^13^C labelling might enhance sensitivity of NMR-based cell culture analyzes. However, NMR spectroscopy represents a suitable tool to monitor ECM remodelling characteristics in FCAVD progression in diseased tissue and in in vitro calcification models.

## Materials and methods

### Human tissue

FCAVD tissues were obtained from patients undergoing aortic valve replacement for severe AS. The study was approved by the local Ethical Committee (Medical Faculty, University Leipzig, registration number 128/19-ek) and all patients gave written informed consent in accordance with the Declaration of Helsinki. All samples showed macroscopic calcification as a sign of progressed FCAVD. Five non-diseased tricuspid aortic valves were removed from body donors (kindly provided by the Institute of Anatomy of the University of Leipzig (CT, IRB 129/21-ek)).

### VIC isolation and cultivation

FCAVD tissue obtained from aortic valve replacement was washed immediately in phosphate-buffered saline (PBS) and was scratched with a razor blade to remove endothelial cells. Leaflets were cut into small fragments of about 1 mm^3^, transferred into 15 ml serum-free Dulbecco's Modified Eagle Medium (DMEM; Gibco) containing 1 mg/ml collagenase (*Clostridium histolyticum*; Sigma-Aldrich) and incubated for 60 min at 37 °C with repeated shaking. Subsequently, tissue fragments were washed with DMEM and the supernatant with remaining endothelial cells was discarded. The tissue was immersed in collagenase solution and digestion was maintained for another 180 min, before the mixture was mixed, filtered through a 40 µm cell strainer and centrifuged at 2000 rpm for 7 min. The cells were cultivated in DMEM containing 10% fetal bovine serum (FBS; PAN Biotech) and 1% penicillin/streptomycin (P/S; Sigma Aldrich) up to a confluency of 90%. All experiments were performed in passages 3–4.

VICs were cultivated up to a 100% confluency and then assigned to: (i) cultivation in control medium (DMEM, + 5% FBS and + 1% P/S) or (ii) cultivation in PM (CM + 50 µg/ml l-ascorbic acid (Sigma-Aldrich) + 2 mM sodium phosphate (NaH_2_PO_4_; Sigma-Aldrich)). The medium was exchanged every 2–3 days. After 21 days, cell samples were washed twice with distilled water and harvested for further analysis.

### Alizarin red staining and quantification

Alizarin red staining was used to visualize and quantify in vitro calcification. The cells were washed with 1 × PBS, fixed with 10% formaldehyde for 15 min, washed twice with distilled water, incubated with 2% Alizarin Red solution (LifeLine Cell Technology) for 20 min at room temperature and finally washed three times with distilled water to remove excess stain. Then 100 mM hexadecylpyridinium chloride (Sigma-Aldrich) was added and incubated for three hours at room temperature before absorbance was measured at 540 nm (Infinite 200Pro, Tecan i-control) to quantify the Alizarin red staining.

### Solid-state NMR spectroscopy

^13^C and ^31^P MAS NMR measurements were performed on a 700 MHz Bruker Neo NMR spectrometer equipped with 3.2 mm E-free and 3.2 mm HFX probes. Valve tissue was homogenized by crushing in liquid nitrogen as previously described^25^. Samples were transferred into a 3.2 mm rotor including a small piece of polyoxymethylene (POM) serving as internal standard.

^13^C MAS NMR experiments were performed at 4 °C and an MAS frequency of 10 kHz. For ^13^C NMR spectra with direct excitation 13,312 scans were recorded using an Hahn echo sequence with a 4 µs long 90° excitation pulse for ^13^C, low power proton decoupling (Spinal64) during acquisition with a field strength of 6.25 kHz and a recycle delay of 5 s. For CP (excitation of ^13^C nuclei via dipolar couplings from spatially close protons) ^13^C NMR spectra, 8192 scans with a 90° ^1^H excitation pulse of 4 µs length, a CP contact time of 0.7 ms with a ^1^H CP spin lock field of 51.5 kHz and a recycle delay of 2.3 s were collected under Spinal64 decoupling of ω_H_/2π = 62.5 kHz during detection. Spectra were processed using Topspin 4.0.9 with an exponential line broadening of 50 Hz and 200 Hz for directly excited and CP NMR spectra, respectively. For figures, spectra were scaled using the sample weight without the internal standard to yield an intensity per mg wet sample, to allow visual inspection. For semi-quantitative assessment of the collagen content, the intensity of the Hyp C_γ_ resonance in the CP NMR spectrum as well as intensity of the POM was used as described before^39^.

^31^P NMR spectra were acquired at 4 °C and 20 kHz using a 4 µs 90° excitation pulse for ^1^H or ^31^P. For directly excited NMR spectra, 40 scans with Hahn echo excitation and a recycle delay of 400 s were acquired with a ^1^H decoupling field strength of 28 kHz. ^31^P CPMAS spectra had a recycle delay of 4 s, a contact time of 0.7 ms, a ^1^H spin lock field of 62.5 kHz and a decoupling field of 28 kHz during acquisition of the 512 scans. NMR spectra were processed with an exponential line broadening of 200 Hz and scaled as described for the ^13^C NMR spectra. Integration of the directly excited spectra was performed for quantification as the receiver gain and number of scans were kept constant during measurements. As quality control, a reference specimen consisting of HAP nanoparticles was measured weekly to ensure stable sensitivity of the spectrometer. These four measurements of the control substance yielded signal integrals in the directly excited spectra with a maximal deviation of 3.9% of the mean value, allowing quantification without internal standard.

### Statistical analyses

Data was analyzed using two-sided t-test (GraphPad, PRISM, V8). Statistical analysis of the collagen and hydroxyapatite content measured by NMR was performed using a two tailed Mann–Whitney *U* test. All *p*-values < 0.05 were considered statistically significant.

### Supplementary Information

Supplementary Figures.

## Data Availability

The datasets generated and/or analysed during the current study are available from the corresponding author on reasonable request.

## Abbreviations

AS: Aortic valve stenosis
CM: Control media
CP: Cross polarization
CT: Control tissue
ECM: Extracellular matrix
FCAVD: Fibro-calcific aortic valve disease
GAG: Glycosaminoglycans
HAP: Hydroxyapatite
Hyp C_γ_: Hydroxyproline C_γ_
MAS: Magic angle spinning
NMR: Nuclear magnetic resonance
PC: Phosphatidylcholine
PE: Phosphatidylethanolamine
PG: Phosphatidylglycerol
PL: Phospholipids
POM: Polyoxymethylene
PM: Procalcifying media
SNR: Signal-to-noise ratio
TAG: Triacylglycerols
VIC: Valve interstitial cell
