# Supplementary material for: Nuclear magnetic resonance spectroscopy to quantify major extracellular matrix components in fibro-calcific aortic valve disease
Source: Sci Rep. 2023 Nov 1;13:18823. doi: 10.1038/s41598-023-46143-7 (PMC10620231; doi:10.1038/s41598-023-46143-7)
Supplement: Supplementary file 1 — Supplementary Figures. [file 41598_2023_46143_MOESM1_ESM.docx]

**Supplemental material:**


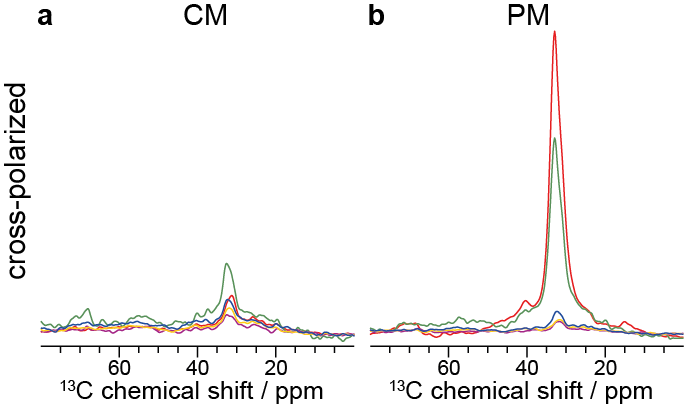


**Fig. SI1.** ^13^C CP NMR spectra of VICs treated with control media (CM, **a**) or procalcifying medium (**b**) after 21 days. Spectra are scaled per mg wet sample weight for better comparison.


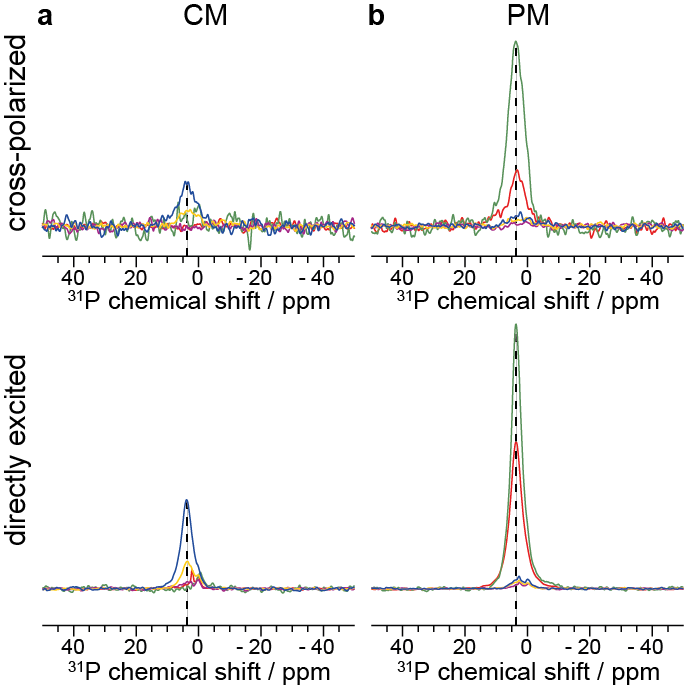


**Fig. SI2.** ^31^P CP NMR spectra (upper panel) and ^31^P directly excited NMR spectra (lower panel) of valve interstitial cells after 21 days of treatment with control medium (**a**, left column, CM) and treatment with pro-calcifying medium (**b**, right column, PM) to induce mineralization. Spectra are scaled per mg wet sample for better comparison.
